# Prophylaxis of Pain and Fractures within Feet in the Course of Osteoporosis: The Issue of Diagnosing

**DOI:** 10.1155/2020/1391026

**Published:** 2020-11-29

**Authors:** Aleksandra Bitenc-Jasiejko, Krzysztof Konior, Kinga Gonta, Magdalena Dulęba, Danuta Lietz-Kijak

**Affiliations:** ^1^Department of Propaedeutic, Physical Diagnostics and Dental Physiotherapy, Pomeranian Medical University in Szczecin, Szczecin, Poland; ^2^Doctoral Study Department of Propaedeutic, Physical Diagnostics and Dental Physiotherapy, Pomeranian Medical University in Szczecin, Medical Center in Nowogard, Szczecin, Poland; ^3^College of Physiotherapy in Wroclaw, Ortogenic Rehabilitation and Podology Center in Wroclaw, Wroclaw, Poland

## Abstract

**Background:**

Considering the enormous risk of fractures in the course of osteoporosis in the area of the feet, an important aspect of prophylaxis is periodic and, in special cases, ongoing monitoring of defects and deformations as well as pressure distribution. The purpose of this article is to indicate the role of the examination of posture and pressure distribution during standing, postural balance, and gait, in the prevention of fatigue fractures in the course of osteoporosis, based on the literature review and examples of patients.

**Methods:**

The manuscript consists of two parts; it has a review-analytical character. The first part reviews the literature. The data were obtained using the MEDLINE (PubMed), as well as Cochrane and Embase databases. The database review was carried out focusing mainly on English-language publications, while taking into account the topicality of scientific and research works in the area of osteoporosis. The problem of multiaspects in the area of bone density was pointed out. Considering the above, in the second part, the authors analyzed 11 exemplary patients with osteoporosis, referring to the assessment of foot and lower limb defects using traditional posturological methods and including pedobarography to diagnostic procedures that are used in the assessment of pressure distribution, standing and moving, and an attempt to balance.

**Results:**

Analysis of the research and scientific literature proved the lack of unambiguous diagnostic procedures of the locomotor system recommended for the prevention of fatigue fractures in the course of osteoporosis. The main diagnostic recommendations are imaging tests (most often X-ray), which are recommended in the case of specific clinical symptoms. The analysis of exemplary patients with osteoporosis showed numerous disorders in the distribution of pressure in the plantar part of the feet, which are related, among other things, with their individual defects and lower limbs.

**Conclusions:**

Detailed posture diagnostics and gait estimation, along with the analysis of pressure distribution within the feet are a very important aspect of the prevention of structural degradation and fatigue fractures within the feet. An important postulate for further research and scientific work is the elaboration of the procedures that will serve the preventive diagnostics of the locomotor system, aimed at early detection of threats of fatigue fractures.

## 1. Introduction

Osteoporosis is defined by the World Health Organization (WHO) as a reduction in the bone density index, which affects about 30% of women and 12% of men [1]. The issue of osteoporosis depends mainly on its differentiation, especially on the problem of bone osteomalacia, which has similar causes. Equally, in osteoporosis and osteomalacia, as well as in osteopenia, the most common element of these diseases is vitamin D deficiency, which leads to impaired phosphate and calcium concentrations. Vitamin D is produced inside the skin due to the influence of sunlight and supplied to the body with nourishment. Osteomalacia may also be caused by calcium and phosphate insufficiencies, for reasons unrelated to vitamin D (e.g., dietary deficiencies), lack of exposure to the sun, and malabsorption of vitamin D from the digestive tract or kidney and liver failure [2–5]. The analysis of osteomalacia usually detects decreased concentrations of calcium (may also be regular), phosphates, and the active form of vitamin D (25-OH-D), as well as an elevated concentration of alkaline phosphatase (ALP) [6]. In the definition of osteoporosis, WHO defines a reduced T-score of bone mineral density (BMD) as-2.5 (T-score < −2.5), diagnosed by X-ray imagining double emission (measured by dual-emission X-ray) [7–11]. Innovative medical engineering methods, the so-called Nanoindentation, indicate that there are significant contrasts in bone hardness in patients with osteoporosis, which affects their load resistance, deformation possibilities, and flexibility, while reducing microhardness in relation to the control group [12, 13].

Bone fragility and, as a result, fractures of the distal part of the foot, pelvic bone, sacral bone, head of the proximal tibial bone, and ribs may be a manifestation of osteomalacia, with a deficiency of vitamin D3 and an equally abnormal absorption of calcium ions and phosphates. These issues should be differentiated from fractures due to osteoporosis in which there is a rupture of the femoral neck, spine vertebrae, or spondylopathy by reducing bone mineral density, which are determined by densitometric examination. The fracture risk increases with age [1, 14, 15].

Since 2001, osteoporosis has been ultimately defined as a disorder within the skeletal system, in which the risk of bone fracture increases, not only due to their reduced density, but also in the course of their fragility, which rises with age [16]. The lack of calcium or phosphates leads to decreased bone mineralisation, while a deficiency of vitamin D leads to weakened muscle receptors [17, 18]. Hypophosphatemia is also induced by drug intake, for example, neutralising phosphates or diuretics, and also, although less frequently, by hereditary diseases and paraneoplastic production of phosphatonins. Additionally, this may be caused by conditions such as prostate cancer and bronchial cancer [19–22]. Therefore, it should be noted that osteoporosis is often a secondary disease in patients with chronic kidney, liver, and lung diseases, often resulting from ageing. Idiopathic osteoporosis also represents an essential aspect of considerations regarding the prevention of bone fractures and, as a consequence, the therapeutic challenge of an unknown etiological factor [23].

Osteoporosis is more common in women, and an additional factor determining its occurrence is menopause and the postmenopausal period (PMOP: postmenopausal osteoporosis), which is related to the impact of oestrogen on osteoclasts [24–29]. It has also been demonstrated that the number of pregnancies, age, and body mass index increase the risk of osteoporosis [30]. However, a much more important determinant of the fractures arising in the course of osteoporosis is advanced age [31]. Microlesions of tissues develop over time; nonetheless, in elderly women, fractures and overload cracks are more frequent [15, 32, 33]. It has been proven that age, BMI, and number of pregnancies are important determinants of the development of postmenopausal osteoporosis [34]. The fundamental and recurrent causes of decreased BMD index in the elderly, apart from systemic diseases and medications intake, include the improper supply of nutrients and a lack of physical activity [35–37].

Osteoporosis is a significant issue in diabetic patients, especially in terms of the prevention of diabetic foot syndrome and defragmentation, as well as bone fractures in the course of Charcot neuroosteoarthropathy. In the course of type I diabetes mellitus, a mild reduction of bone mineral density is noticed, whereas in type II diabetes mellitus, which is more frequent in older people, a regular or increased BMD index is observed. An enhanced risk of fractures in individuals with diabetes is associated with peripheral neuropathy and weakened muscle capacity, which in consequence affects local structural instability caused by hyperglycaemia, particularly glycosylation in collagen fibres [38]. Permanent degradation of the osteoarticular structures of the foot is commonly caused by chronic, often multiple joint inflammation. An additional factor is the instability of the body's balance, which increases the danger of falling. As a result, osteoporosis in diabetic patients is a secondary disease in coexisting kidney diseases, angiopathy, and so on. Research in patients with type I diabetes indicates an elevated number of fatigue fractures [39–44].

Furthermore, it should be noted that the relationship between diabetes mellitus and osteoporosis is imprecise, while bone density studies in the course of diabetes often do not present clear outcomes [45]. Nevertheless, considering the great importance of fracture prophylaxis in diabetic patients, which is strongly influenced by bone mineralisation, bone density, and elasticity, the authors believe that taking into account individual assessments of the coexistence of osteoporosis is a crucial priority. The studies show that over 50% of the general population with diabetes and 20% of younger people (aged 20–56) burdened with the disease fulfil the criteria of osteoporosis [46, 47]. The examination of patients at risk of Charcot's neuroosteoarthropathy is of particular importance, as, in these individuals, the bone mineral density has been significantly reduced [48]. It should be noted that within the feet there are relatively few cross-sectional studies on bone density, mineralisation, and so on.

On the internal level, the function of mechanical load detection is carried out by osteocytes [49–52]. Biomechanisms, aimed at the reconstruction of the bone structure and repair of microdamage, cause periodic bone-weakening at the resorption site. During healing, this particular bone area (adhesion process) shows a periodically elevated fracture risk [53, 54].

On the external level, the key issue in the elimination of biomechanical abnormalities will be the diagnosis of body posture, including diagnostic methods aimed at the early detection of defective pressure distribution during standing, body balancing, and locomotion. As a consequence, these actions are aimed at defect correction, relief, and amortization. As a part of the prevention of fractures, pharmacological treatment, supplementation of vitamin D and calcium, and diet therapy are mainly recommended [55–57]. An important aspect is also the monitoring and treatment of comorbidities, that is, those that constitute the main cause of osteoporosis [58, 59]. Diagnostics of the musculoskeletal system is recommended mainly to patients at risk, mainly the elderly [60] and women in the postmenopausal age [61, 62]. However, for younger people, routine tests are recommended [60]. Detailed imaging diagnostics is performed in the event of emerging clinical symptoms (pain, inflammation, and swelling). The most frequently recommended examination is X-ray, which, unfortunately, in the initial phase shows a low diagnostic sensitivity, increasing after about 3 months from the appearance of the first symptoms [63–68].

However, research and the scientific literature do not indicate detailed noninvasive diagnostic procedures. There are also no scientific and research reports in the area of foot assessment, including in particular the procedures for assessing the distribution of pressure within the feet.

From the authors' experience, the diagnosis of postural and functional defects of structures allows the early detection of the risk of fatigue fractures in patients with osteoporosis. Consequently, periodic tests allow the early implementation of effective measures to prevent fatigue fractures. Such activities are of particular importance in the prevention of foot fractures, mainly due to the significant gravitational overloads arising during locomotion. Considering the above, the authors made a detailed diagnosis of posture in 11 exemplary patients with osteoporosis, indicating at the same time diagnostic methods for the assessment of foot defects and deformities, and the analysis of pressure distribution during standing, walking, and body balancing.

The effects of the analysis of exemplary patients.

All figures and images have been prepared by the authors and are their property.

The analysis of patients included 11 exemplary patients with osteoporosis, aged 27–86, including eight men and 3 women with pain in the feet, ankle joint, and/or shin, with a limited range of mobility. Two patients additionally suffer from diabetes; four of them have a history of fatigue fractures. In all these patients, postural diagnostics was performed, with particular emphasis on the assessment of foot and lower limb defects using traditional posturological methods, including pedobarography in diagnostic procedures, which is used to assess the distribution of pressure in the standing and locomotion position and the balance test.

Table 1 presents the results of tests of 11 patients, indicated in this publication, and photogrammetric research, which were accompanied by anthropometry of the foot and podoscopic tests in individual sectors of the foot including the following:

  Arch height measurements that have been combined with the plantar part assessment
  Goniometric assessment of the walk angle and toes position

The results of the examinations of the feet performed in patients with osteoporosis indicate that 9 out of 11 suffer from tarsal valgus. Seven patients presented hallux valgus and defects of the toes from II to V. However, the comparison of the results of the X-ray with the outcomes of the tests indicates that each patient has a different problem and, as a consequence, a different cause of pain and distortion. The clinical exemplary patients presented in this publication show asymmetrical and chaotic dysfunctions of the foot and structural instability. Given the fact that patients have individually specific changes, the diagnostic procedure indicated by the authors points to the necessity of individual determination of the needs in terms of relief, shock absorption, and correction to be performed in patients with osteoporosis.

Below are photographic documentation of 9 patients, that is, X-ray, magnetic resonance imaging, and podoscopic research, which used selected elements of functional diagnostics and anthropometry.

During X-ray tests, hallux valgus with an overload of the metatarsophalangeal joint in the area of MTP I and frequent subluxation of sesamoid bones was observed in five patients. Among four patients, enthesopathy of the plantar fascia was detected. In the case of 4 exemplary patients, fractures within the foot bone were observed, in one exemplary patient case tibial, and fibula bone fractures occurred. The analysis of X-rays shows numerous overload changes in the region of the foot, which is manifested by calcifications and osteophytes in the joints and defective trabeculation.

**Figure 10:**
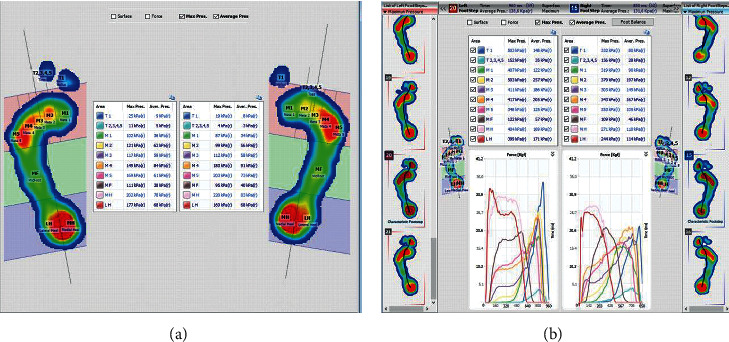
(a) The result of pedobarographic test when standing. (b) While walking, patient no. 10, increased pressure on the M1-M5 metaplanes.

The assessment of the pressure on the arch of the foot when standing and walking will be crucial in the early detection of increased pressure. For this purpose, a pedobarographic test was carried out under both static and dynamic conditions (Figure 10(a). Evaluation of pressure during standing/Figure 10(b): assessment of pressure during walking).

The analysis included the values of pressure in individual metaplanes, determined according to the model, and markings indicate pressure on individual sectors of the foot:

  MH—internal part of the tarsus/LH—external part of the tarsus: allows the reading of pressure, with the correlation of results based on the outcomes of anthropometric tests of valgus/varus tarsus deformity/tarsus instability, both while standing and walking
  MF—metatarsus: allows the pressure on the metatarsus to be assessed; this is especially important in assessing pressure during walking
  M 1-5—pressure on the metatarsophalangeal joints: allows conclusions to be drawn on the functionality of the transverse arch
  T1—pressure on the great toe and T2-5—pressure on the toes 2–5

The results of the examination show a significant overload of the forefoot, with fractures and degradation of the 3rd metatarsal head (M3 meta surface). It is noteworthy that the compensatory processes that reduce pain while standing do not activate while walking (despite persistent pain), while standing the foot is stabilized by the tarsometatarsal joints (TMTJ), which consequently makes the second and third metatarsal bones susceptible to fractures as a result of stress [69–71].

Patient no. 10 presented adduction and varus deformity of the forefoot at the level of the line of Lisfranc joints. This is also illustrated by the result 10a, in the left foot:

  Increased pressure on the heel in the MH area (which is confirmed by the diagnosis of valgity made in a physical examination).
  Increasing pressure on the head of the 1st (M1) to the 5th (M2) metatarsal bone. This may be due to the fact that metatarsal bones, including the arch of the foot, are exposed to fatigue fractures.

**Figure 11:**
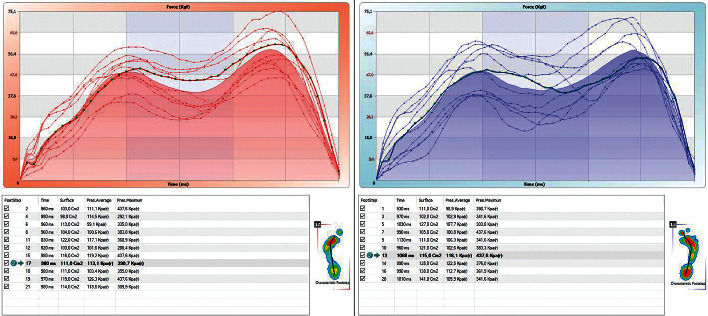
The results of the examination of the distribution of forces and time-space parameters while walking, patient no. 7.

The pedobarographic examination also allowed the assessment of spatial and temporal parameters during foot shunting.  Figure 11 presents the results of the examination of the distribution of forces, pressure, and acceleration for patient no. 7 suffering from a fracture of the calcaneus.

The pressure distribution curve is repeatable, an increased pressure is observed on the forefoot of both feet, and this may be associated with a significant dispersion of the values of forces in individual footprints. This result may indicate instability of the structures of the upper and lower ankle.

**Figure 12:**
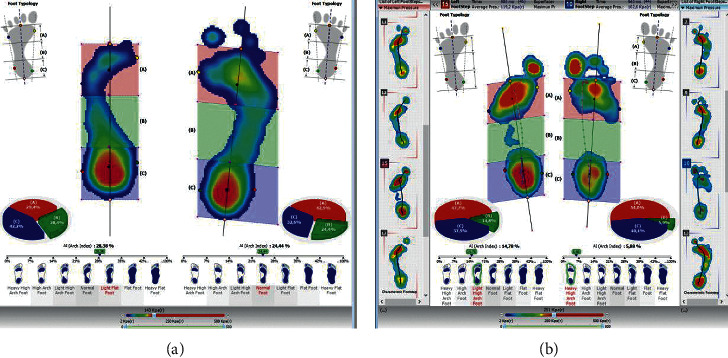
(a) The result of the foot arch examination when standing. (b) The result of foot arch examination while walking: patient no. 7.

The functionality of the longitudinal arch is assessed using pedobarography. A degree of arch arcing while standing is determined using the Biomech Studio software and the AI (Arch Index). The results presented in Figures 12(a) and 12(b) for patient no. 7 confirm instability of the foot structures, because selected traces while walking show asymmetrical and unique overpronation of the foot.

**Figure 13:**
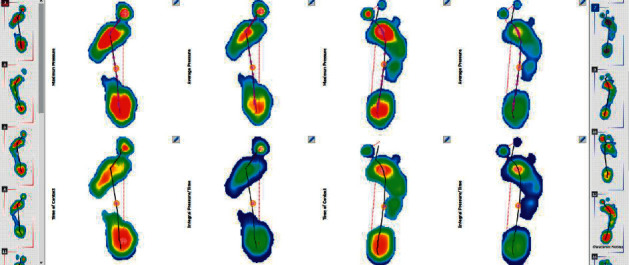
The assessment of the progression of the condition for patient no. 7: push off curve; the thin red line depicts overpronation propulsion of the left foot.

Comparative analysis of the results of foot arch examinations during standing and walking is used to assess the functionality of the feet. Six patients who are presented in this publication have overpronation of the foot, which allows a conclusion of planovalgus foot during walking to be drawn (i.e., lowering of the arch of the foot during the phase of push off and no lateral edge contact, i.e., no supination of the feet). The push off curve was also observed by pedobarography, as illustrated in Figure 13.

**Figure 14:**
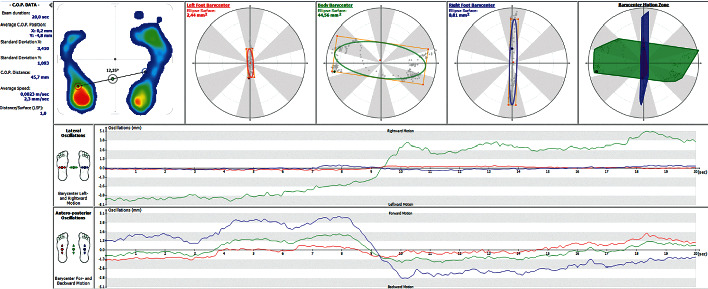
The result of the balance test for patient no. 7.

The ability to maintain balance, which is also assessed during a pedobarographic examination, is also important in assessing the distribution of pressure on the feet.  Figure 14 shows the result of the balancing test in patient no. 7. The result indicates a significant imbalance, especially for the lateral oscillations of the COP (Figure 14).

The pedobarography has a very wide diagnostic spectrum, while both standing and walking. Therefore, it is widely used in the prevention of fractures resulting from overloads. These include, in particular, osteoporosis.

## 2. Discussion

In the prophylaxis of secondary fractures, which occur during the curing process, it is essential to create the appropriate conditions through two factors:

- Elimination of improper pressure, through depreciation and relief
- Targeting of its accurate distribution

These factors are strategic goals in the process of reducing clinically visible changes [37, 72]. In the course of the diagnostics and therapy, the time periods of the applied procedures should be taken into account. This emphasises the fact that after the reconstruction phase in patients with osteoporosis, the bone structure has an extended mineralisation time [12, 73].

Reduced bone density and weakened mechanisms of its reconstruction, repair of microdamage, defects in the microarchitecture of the trabeculae, and the porosity and thinning of the cortical layer require rapid prophylactic actions, starting at the level of loads arising in the course of everyday life [74–77]. However, this does not indicate the exclusion from activity, as it has been proven that immobilisation does not promote bone remodelling [78–80]. The skeletal system has an autoprogramme, targeted at the thinning of unused bone, which was observed in military workers as well as professional athletes. Thus, raising the load on the bone raises its weight. Nevertheless, it should be emphasised that improper pressure distribution may promote the local accumulation of microdamage. In the case of an incorrect tension level, the process of decreasing/increasing of bone mass may result in microdamage and, in the long term, may cause morphological changes of bones, which through a loss of elasticity may often result in noticeable fractures [51, 81–83]. This phenomenon particularly concerns the foot, because its function, apart from locomotion, is postural stabilisation when standing and walking. The feet accommodate to the surface changes and movements performed by a person, changing the length of soft structures and the arrangement of hard ones and then subsequently returning to their architecture at the moment of relief. Another function of the foot is to act as a shock absorber, which not only exposes it to numerous overload changes but also to microinjuries resulting from excessive impacts and stress pressure [84–86].

In the assessment of the foot's condition, and especially in the activities focused on the prevention of overload changes, the diagnosis of its dynamic parameters and support function has a significant role. Adjusting the pressure distribution, carried out in the process of microdamage prophylaxis, should, therefore, be a process of balancing between the motor activity and simultaneous elimination of excessive load, arising during everyday life activities [87–91]. In a physical activity schedule, an essential role of rest, as well as periodic breaks in training, was indicated, as part of fatigue fracture prevention [92]. Antigravitational exercises, such as swimming, will play a significant function in the time of demand for periodical relief and in individuals at risk of fracture formation [93, 94]. The return to regular activity should be strictly supervised and based on an accurate diagnostic-rehabilitation plan [69, 95].

The recommended treatment, as a component of fracture prophylaxis, mainly involves the initiation of pharmacological treatment, after prior bone density diagnosis and/or X-ray examination. Diet therapy, healthy lifestyle, and vitamin D and calcium supplementation reduces the fracture risk; nevertheless, it is suggested to estimate the intake of calcium due to controversies related to its supply and risk of cardiovascular diseases [55–57]. In the therapy of osteoporosis, bisphosphonates are the fundamental pharmacological agents. The remaining drugs are aimed at reducing the risk of nonvertebral and hip fractures, as well as treating systemic diseases that are the main cause of osteoporosis [58, 59]. International guidelines and recommendations for the pharmacological treatment of osteoporosis indicate minor differences, particularly in the age range of patients [11, 61, 96–98]. There are few reports in the research and scientific literature that would indicate an important role in the prevention of fractures and therapy through the use of individual orthopaedic insoles, specialist footwear, orthopaedic cushioning, and relieving elements. In the opinion and recommendations of the authors, it should be extended and include a significant role of relieving, amortization and correcting defects and dysfunctions of the foot, manifested during walking, running, standing, and other life activities.

The research and scientific literature indicates that, in the area of locomotor system diagnostics, as well as in the investigation of fractures in the course of osteoporosis, a highly specialised imaging diagnostic examination remains the golden means (most often, these are X-ray examinations). This is performed in the majority of cases when the patient already reports specific clinical symptoms, such as pain during normal life functions [69]. This situation is frequently the case when a fracture has already occurred. However, there is no fatigue fracture classification system that can be applied to all bones. The estimation schemes, commonly quoted by Fredericson et al. [64], Aredt at al. [65], and Nattiv et al. [66], do not include any recommendations for foot assessment. Although X-ray examinations are easy to perform and relatively inexpensive, they show a low sensitivity (15%–35%) in the initial phase of lesions, which increases about 3 months after the first symptoms appeared [67, 68].

The scientific and research literature does not indicate specific diagnostic procedures. The studies of patients with osteoporosis, reviewed in this publication, showed a chaotic and asymmetrical and highly individualized course of dysfunctions in the area of defects and diseases of the lower extremities. According to the authors, individual differences are closely related to coexisting diseases, in particular to postural defects, with a very individualized course. This is an important postulate for further research aimed at the creation of diagnostic procedures, which are procedures applicable in the prevention of fractures in the course of osteoporosis. The imaging diagnostics recommended so far, due to its intended use, obviously do not constitute preventive procedures for the early detection of fatigue fracture threats. According to the authors, early detection of defects, distortions, gait disturbances, and mobility of structures (including in particular structural instability) will be an important aspect of preventing osteoporotic (and other fractures arising in the course of low bone density) fractures.

In the course of prophylaxis, screening is recommended, including women aged over 65 and men aged over 75, among patients at risk. Recommendations are population studies of older people; younger ones should be routinely assessed [60] and all women after menopause [61, 62]. Within the field of foot diagnostics, the interdisciplinary role of the medical team is crucial [99]. According to the authors, considering only the elderly in the screening diagnosis, and thus examining younger people only in the case of emerging clinical symptoms, significantly reduces the effectiveness of preventive measures.

Preventive actions in the area of fractures in the course of osteoporosis should be focused on precise feet screening diagnostics and, also, due to the fact of a close relationship between the action of forces during everyday life activities and the formation of microfractures, breakages, and a lack of adhesions, in patients with reduced bone density (BMD) [100, 101]. It is estimated also that the occurrence of osteoporotic fractures in the foot is rising, mainly due to the fact of the ageing of the population and the increase within the number of people exercising above 50 years [63]. Another important element determining microdamage and bone fractures is the body mass index (BMI), which applies to each age group, that is, both elderly and adolescents [102–104].

According to the researchers, the diagnosis of pressure distribution through pedobarography is crucial for the prevention of tissue destruction within the foot. The assessment of defects and functions of the feet, especially during locomotion, serves for the precise detection of the causes of faulty distribution of the tensions. The essence is to differentiate the pressure that occurs while standing and walking, mainly due to the compensatory processes that maintain balance [105–107]. Pedobarography also allows for the assessment of the average time-space parameters and pressure forces especially during walking. This is a very important diagnostic aspect; it was assumed that considering that the heel stroke accounts for 110% of body weight and increases to 250% of body weight during running, the pressure distribution on the forefoot increases by about 40%–50% [108, 109]. The prevention of fatigue fractures should include monitoring pressure forces over time and measuring the values of acceleration of the foot during the phase of push off. Disturbances in the distribution and directions of forces are an absolute determinant of overload fractures. Drawing conclusions on pressure distribution disorders is of key importance, in particular in the assessment of the average obtained for a large number of samples, acquired in one patient. As a result of the pedobarographic examination, the result is obtained regarding the foot arch, pronation and supination functions, so it is also possible to infer about the foot structure and function during walking. All these aspects constitute important conclusions regarding the stability of structures, locally and globally (i.e., the balance of the body when standing). Instability of the foot structures not only can cause imbalance during walking, which promotes injury during a fall, but also can be manifested as imbalance when standing. The prevention of fractures should include the prevention of falls, in particular in patients with bone density deficits [11, 61]. In addition, patients at increased risk of falling should be given high doses of vitamin D [57]. The development of osteoporosis is promoted by an overload of the musculoskeletal system, body weight transfer during locomotion (movement), and losing and regaining balance. The lower limbs are particularly overloaded in the exemplary patient [110].

The balance test is an important aspect of functional diagnostics of the musculoskeletal system; combined in one device (i.e., a pedobarograph), it significantly reduces the cost of biomechanical diagnostics [111–118]. Also, when bone structures are injured, both crushing and microfractures are noticed; current observations through noninvasive diagnostic methods and the implementation of relieving measures are of great importance in the prevention of complications, including the avoidance of secondary fractures.

It seems evident that deformities in the feet and lower limbs, and generally defects in posture, will encourage the formation of defective pressure distribution and a point increase in its value. At the same time, whereas the structural instability induced by osteoporosis will favour the occurrence of defects, structural inefficiencies, and balance disorders, we may speak of a kind of “vicious circle” of events. Therefore, according to the authors, the screening of patients with osteoporosis will also be crucial within the following:

  Initial assessment of foot defects, for example, valgus/varus deformity of the tarsus, height of the vault, and deformations of the toes (mallet toes, claw toes, hallux valgus, and varus toes)
  Reliable estimation of pressure distribution under static conditions
  Evaluation of foot progression during walking, running, and other types of locomotion
  Disturbances of the gait determination in terms of defective pressure distribution, including particularly the relationship of increased tension during prolonged contact of the foot with the ground
  The importance of patient education in the field of foot observation and fast intervention in case of local pain, redness, and local body temperature increase [119–121]

Screening tests of feet should consist of the assessment of skin condition for hyperkeratosis, since the first symptoms of increased pressure within the feet are calluses and blisters, formed in the soles of the feet [122, 123]. The analysis of pressure distribution is not only important in prevention and screening, but also an important aspect in planning relief procedures and individual orthopaedic supply [124]. Measurable and precise diagnostic methods of pressure (in point and global terms) and the assessment of time and contact surface of the plantar part of the feet with the ground should be used to design individual orthopaedic insoles and footwear [125, 126]. It has been proven that orthopaedic insoles with individually designed elements of the foot arch (i.e., longitudinal and transverse arch) significantly improved body balance in older women with osteoporosis and were an important aspect of preventing falls, sprains, and so on [127].

The lack of ability to relieve the feet and no autocorrection during gait (supination/pronation) is one of the most important problems of patients with diabetes and its complications, for example, Charcot neuroosteoarthropathy [128]. This is an important aspect due to the fact that osteoporosis quite often statistically coexists with diabetes. It is necessary to implement targeted rehabilitation measures and appropriate relief, corrective and shock-absorbing supply in particular in the prevention of microfractures, ligament damage, and muscle atrophy in the course of sensory neuropathy [129]. The supply should take into account, first of all, important parameters showing foot dysfunction, gait disturbances, defects, and deformations [130–132]. Patient education and support in the selection of footwear with an individual orthopaedic insole are also necessary in the holistic prevention of fractures in osteoporosis. For the exemplary patient, footwear should be tailored to the needs of patients. This is particularly important in the prevention of fractures in patients with severe foot deformities in osteoporosis. These are also important postulates for further research and scientific works planned by the authors.

## 3. Conclusions

1. The analysis of the literature showed that, apart from the diagnosis of bone density and the assessment of vitamin D levels, calcium levels, and so on, highly specialised imaging tests (mainly X-ray), used in the event of clinical symptoms, are the recommended diagnostic procedures in the area of the musculoskeletal system.
2. Patients with osteoporosis show numerous individual deformities (defects), dysfunctions, and structural changes in the feet, which results in various disorders of pressure distribution. Extending diagnostics by periodic and screening tests, focused on assessing foot defects, balance disorders, and monitoring time-space parameters during gait will be an important aspect in the prevention of fractures in the course of osteoporosis.
3. Pedobarography has a wide range of uses in periodic screening and ongoing foot diagnostics when the first symptoms of overload (e.g., corns, calluses, pain, and redness) have appeared.
4. Instability and locally increased pressure are observed especially during locomotion; therefore, it is an important aspect to conduct a detailed analysis of patients while walking.
5. In addition to supplementation and pharmacotherapy, the prevention of osteoporotic fractures should include the use of orthopaedic insoles, taking into account the patient-tailored design of elements to relieve and absorb shocks and correct deformations and defects.

## Figures and Tables

**Figure 1:**
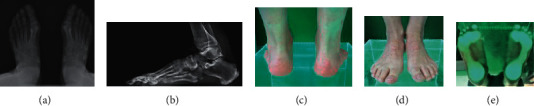
(a–e) Results of imaging tests (X-ray) and podoscopic tests: patient no. 1. (a). Dorsal-plantar foot X-ray: hallux valgus, subluxation of sesamoid bones, overloaded cuneometatarsal joint, and osteophytes in the area of navicular bone.(b) Lateral X-ray: Achilles tendon enthesopathy and numerous calcifications. (c) Posterior picture: ankle joint and hindfoot evaluation. (d) Anterior picture of the feet: assessment of toe positioning and deformation. (e) Plantar photo on the podoscope: evaluation of plantar part of the foot and height measurement of the longitudinal arch.

**Figure 2:**
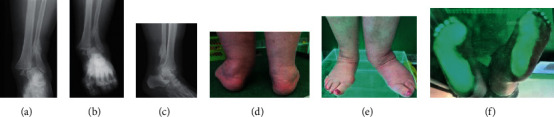
(a– f) Results of imaging tests (X-ray) and podoscopic tests: patient no. 2. (a) Posterior-anterior X-ray; fracture of the medial ankle and fracture of the distal end of the fibula. (b) Anterior-posterior X-ray: fracture of the medial ankle, fracture of the distal end of the fibula, overload changes in the phalangeal joints, and inflammation in the metatarsal joints. (c) Lateral X-ray: microfractures of the heel bone and enthesopathy of the plantar fascia: calcaneal spur. (d) Posterior picture: ankle joint and hindfoot evaluation. (e) Anterior picture of the feet: assessment of toe positioning and deformation. (f) Plantar photo on the podoscope: assessment of plantar part of the foot, height measurement of the longitudinal arch.

**Figure 3:**
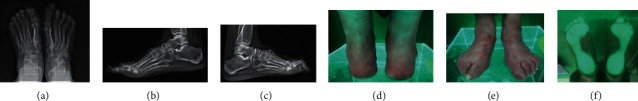
(a–f) Results of imaging tests (X-ray) and podoscopic tests: patient no. 4. (a) Dorsal-plantar foot X-ray: phalangeal valgity, degeneration of the interphalangeal joints, and additional navicular bone in the left foot. (b) Lateral X-ray: coronoid talus bone and degradation lesions in the phalangeal joints. (c) Lateral X-ray: coronoid talus bone and degradation lesions in the phalangeal joints. (d) Posterior picture: ankle joint and hindfoot evaluation. (e) Anterior picture of the feet: assessment of toe positioning and deformation. (f) Plantar photo on the podoscope: assessment of plantar part of the foot and height measurement of the longitudinal arch.

**Figure 4:**
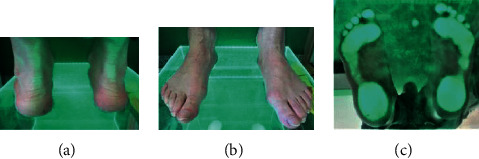
(a–c) Podoscopic tests: patient no. 5. (a) Posterior picture: ankle joint and hindfoot evaluation. (b) Anterior picture of the feet: assessment of toe positioning and deformation. (c) Plantar photo on the podoscope: assessment of plantar part of the foot and height measurement of the longitudinal arch.

**Figure 5:**
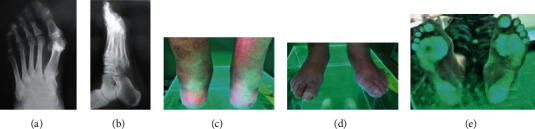
(a–f) Results of imaging tests (X-ray) and podoscopic tests: patient no. 6. (a) Dorsal-plantar foot X-ray: degeneration of the I head of metatarsal bones, phalangeal valgity, and overload lesions in metatarsophalangeal joints. (b) Lateral foot X-ray: enthesopathy of the plantar fascia and inflammation in the metatarsophalangeal joints. (c) Posterior photo: ankle joint and hindfoot evaluation. (d) Anterior picture of the feet: assessment of toe positioning and deformation. (e) Plantar photo on the podoscope: assessment of plantar part of the foot and height measurement of the longitudinal arch.

**Figure 6:**
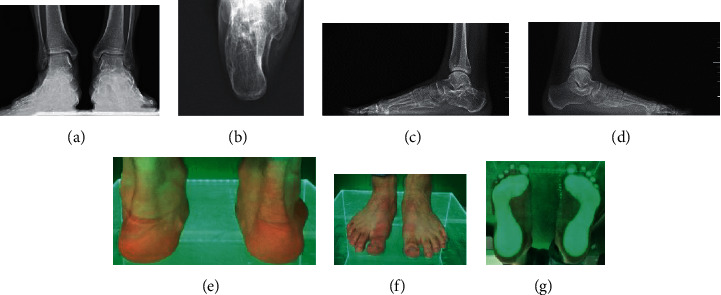
(a–g) Results of imaging tests (X-ray) and podoscopic tests: patient no. 7. (a) Anterior-posterior X-ray, numerous overload changes in the upper ankle joints, tarsometatarsal joints, and calcifications. (b) Axial X-ray: heel bone fracture. (c) Lateral X-ray: numerous overload changes and calcifications. (d) Lateral X-ray: numerous overload changes and calcifications. (e) Posterior photo: ankle joint and hindfoot evaluation. (f) Anterior picture of the feet: assessment of toe positioning and deformation. (g) Plantar photo on the podoscope: assessment of plantar part of the foot and height measurement of the longitudinal arch.

**Figure 7:**
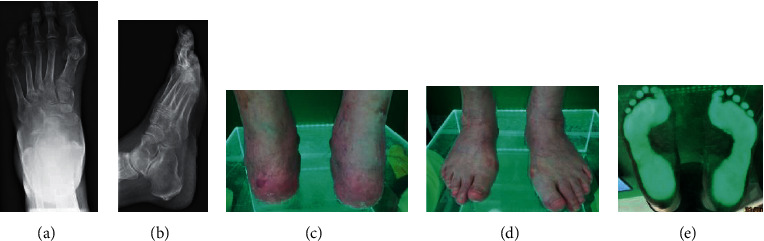
(a–e) Results of imaging tests (X-ray) and podoscopic tests: patient no. 8. (a) Dorsal-plantar X-ray: hallux valgus, subluxation of sesamoid bones, and overloaded cuneometatarsal joint. (b) Diagonal foot X-ray (lateromedial): enthesopathy of the plantar fascia and Achilles tendon, numerous calcifications, and overloads in the upper ankle joint. (c) Posterior picture: ankle joint and hindfoot evaluation. (d) Anterior picture of the feet: assessment of toe positioning and deformation. (e) Plantar photo on the podoscope: assessment of plantar part of the foot, height measurement of the longitudinal arch.

**Figure 8:**
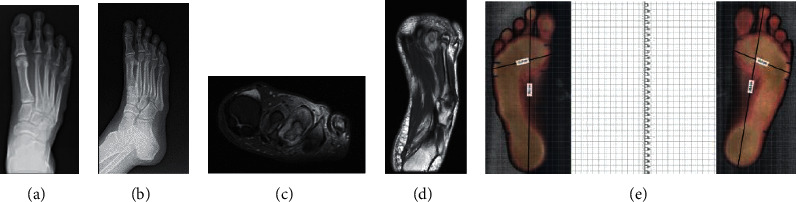
(a–e) Results of imaging tests (X-ray, MRI) and foot scanning: patient no. 10. (a) Dorsal-plantar X-ray: degradation lesion of the III head of the metatarsal bone and numerous transverse calcifications. (b) Diagonal X-ray of foot 45: degradation lesion of the III head of metatarsal bone and numerous calcifications. (c) Magnetic resonance imaging with contrast: degradation lesion of the III head of the metatarsal bones and medial plantar nerve neuralgia. (d) Magnetic resonance imaging with contrast: degradation lesion of the III head of the metatarsal bone and medial plantar nerve neuralgia. (e) Plantar photo from 2D Scanner: assessment of plantar part of the foot and measurement of foot length and width.

**Figure 9:**
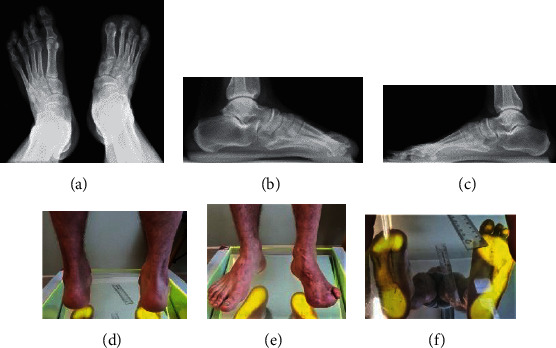
a–f) Results of imaging tests (X-ray) and podoscopic tests: patient no. 11. (a) Dorsal-plantar foot X-ray: fatigue fracture of the III metatarsal bone, amputation of toes I–IV in the left foot. (b) Lateral X-ray: amputation of toes I–IV, overload in the area of ankle joints, and talonavicular. (c) Lateral X-ray: overload in the area of ankle joints and talonavicular. (d) Posterior photo: ankle joint and hindfoot evaluation. (e) Anterior picture of the feet: assessment of toe positioning and deformation. (f) Plantar photo on the podoscope: assessment of plantar part of the foot and height measurement of the longitudinal arch.

**Table 1:** Summary of general information, defects, and ailments of the feet of patients included in the study, together with an assessment of functionality in individual areas of the foot (foot, longitudinal arch, transverse arch, and toes).

Patient no./ figure no.	Medical history			Diagnosed foot defects while standing			
	Age (years)	Weight (kg)	Medical history, complaints	Tarsus	Longitudinal arch	Transverse arch	Toes
No. 1 Figures 1(a)–1(e)	69	70	Pain in the forefoot, both right and left. Limited mobility in the upper ankle joint	L-valgus R-normal	L-reduced R-normal (planovalgus foot in the gait assessment of both feet-pedobarography)	L-reduced R-reduced	L-normal R-normal
No. 2 Figures 2(a)–2(f)	86		Comminuted fracture of the fibula, tibia	L-post fracture valgity R-valgity	L-reduced R-reduced	L-reduced R-reduced	L-hallux valgus R-hallux valgus
No. 3	66	65	Ankle swelling, foot pain	L-valgity R-valgity	L-normal R-normal (planovalgus foot in the gait assessment of both feet-pedobarography)	L-reduced R-reduced	L-normal R-normal
No. 4 Figures 3(a)–3(f)	55	85	Pain of the whole foot hindering walking, venous insufficiency	L-varus deformity R-varus deformity	Varus forefoot-instability at the level of the tarsometatarsal joints	L-reduced R-reduced	L-hallux valgus, II-V-hammer toes R-hallux valgus, II-V varus toes (fixed lesions-X-ray)
No. 5 Figures 4(a)–4(c)	58	95	Foot pain	L-valgity R-valgity	L-normal R-normal (planovalgus foot in the gait assessment of both feet-pedobarography)	L-reduced R-reduced	L – hallux valgus, flexible hammer toes– hallux valgus, flexible hammer toes
No. 6 Figures 5(a)–5(e)	71	91	Pain in the area of the first metatarsophalangeal joint, limitation of mobility. Diabetes II, renal failure, psoriasis, prostate hypertrophy, hypertension, heart failure, fatty liver, lymphoedema	L-valgity R-valgity	L-normal R-normal (planovalgus foot in the gait assessment of both feet-pedobarography)	L-reduced R-reduced overload change (callus) in the area of II and III metatarsophalangeal joint	L-hallux valgus/rigid II, IV varus toes/II hammer toe P-II-IV flexible hammer toes
No. 7 Figures 6(a)–6(g)	27	87	Fracture of the calcaneus with fragmentation in 2015	L-valgity R-valgity	L-normal P-normal (planovalgus foot in the gait assessment of both feet-pedobarography)	L-reduced R-reduced	L-normal R-normal
No. 8. Figures 7(a)–7(e)	79	68	Pain of the left foot, of the great toe, psoriasis	L-valgity R-valgity	L-reduced R-reduced (planovalgus foot in the gait assessment of both feet-pedobarography)	L-reduced R-reduced	L-hallux valgus, dislocation of the sesamoid bones (X-ray)R-hallux valgus
No. 9.	82	94	Significant valgity and pain of the ankle of the left limb; in the right limb, the condition is complicated by surgery due to hallux valgus, lymphoedema, no X-ray-the patient does not agree to the X-ray image	L-valgity (the lack of X-ray image prevents making diagnosis)R-valgity	L-reduced R-reduced(pedobarography)	L-reduced R-reduced	L-hallux valgus, II-IV hammer toesR-significant deformities of the toes (postoperative complications)
No. 10. Figures 8(a)–8(e)	33	87	Pain of the II metatarsophalangeal joint of the left foot, instability of the III toe, microfractures within the head of the III metatarsal bone; medical history revealed numerous microfractures in the right hand, radius, fibula-during activities of daily living (e.g. abnormal body position)	L-normal R-normal	L-normal R-normal Adduction of the metatarsal bones, distortion at the level of the line of lisfranc joints (X-ray, pedobarography indicates significant pressure on the base of the V metatarsal bone). When standing, varus forefoot in relation to the tarsus (pedobarography)	L-reduced R-reduced in both feet, significantly increased pressure on the II metatarsophalangeal joint (pedobarography) degradation of the head of the II metatarsal bone of the left foot (X-ray)	Toes (L and R) apparent features of the Morton's foot caused by adduction of the forefoot (X-ray)II-V flexible hammer toes. Significant instability of the II toe of the left foot
No. 11 Figures 9(a)–9(f)	66	82	L-amputation of the toes I-IV, the metatarsophalangeal joints, as a result of necrosis (circulatory disorders), with an episode of coma, pain of the plantar surface			L-reduced R-reduced	L-amputation of toes I-IV, varus fifth toe (X-ray) P-hallux valgus, II-V flexible hammer toes

L: left limb; R: right limb.
